# Patient-Tailored Aortic Valve Replacement

**DOI:** 10.3389/fcvm.2021.658016

**Published:** 2021-04-21

**Authors:** Ole De Backer, Ivan Wong, Ben Wilkins, Christian Lildal Carranza, Lars Søndergaard

**Affiliations:** ^1^Department of Cardiology, Rigshospitalet Copenhagen University, Copenhagen, Denmark; ^2^Department of Cardiothoracic Surgery, Rigshospitalet Copenhagen University, Copenhagen, Denmark

**Keywords:** aortic valve disease, replacement, repair, surgery, transcatheter

## Abstract

Contemporary surgical and transcatheter aortic valve interventions offer effective therapy for a broad range of patients with severe symptomatic aortic valve disease. Both approaches have seen significant advances in recent years. Guidelines have previously emphasized ‘surgical risk’ in the decision between surgical aortic valve replacement (SAVR) and transcatheter aortic valve replacement (TAVR), although this delineation becomes increasingly obsolete with more evidence on the effectiveness of TAVR in low surgical risk candidates. More importantly, decisions in tailoring aortic valve interventions should be patient-centered, accounting not only for operative risk, but also anatomy, lifetime management and specific co-morbidities. Aspects to be considered in a patient-tailored aortic valve intervention are discussed in this article.

## Introduction

Aortic valve disease—both aortic stenosis (AS) and aortic regurgitation (AR)—represents an important global health problem. Data on the exact prevalence of AS and AR in the general population are lacking, but studies in Western populations estimated that 3–5% of the adult population suffer from moderate or severe aortic valve disease. The prevalence of AS and AR increases with age and it has been estimated that 1% of the population aged < 55 years and 6% of the population aged > 75 years suffer from moderate or severe AS or AR (1, 2). Accordingly, a broad array of strategies to intervene on the aortic valve have been developed.

Aortic valve disease requires mechanical intervention, with medical therapy playing mainly a supportive role and not being able to alter prognosis significantly. The diseased aortic valve can either be replaced or repaired in an open surgical procedure, which for decades has proven to be an effective therapy. More recently, a rapid expansion of transcatheter aortic valve replacement (TAVR) has been noted. Both strategies have their own strengths and weaknesses (3, 4).

Surgical aortic valve intervention encompasses a wide variety of procedures, from a Ross procedure over a surgical aortic valve replacement (SAVR) with root replacement and coronary ostial reimplantation to a more minimalistic aortic valve repair. TAVR is typically less invasive than surgery but offers a relatively limited range of interventional options. A direct comparison of SAVR and TAVR has been made in large randomized clinical trials. Initially, the PARTNER 1 and CoreValve High Risk trial showed the feasibility of TAVR treatment in patients with a high surgical risk (5, 6). More recently, lower risk TAVR trials showed that TAVR can be a good alternative in selected patients with an intermediate to low surgical risk (7–11). Randomized clinical trials comparing SAVR with TAVR showed that both strategies are reasonable options across all surgical risk categories, with available longer-term comparative data up to 8-years of follow-up.

**Figure 1:**
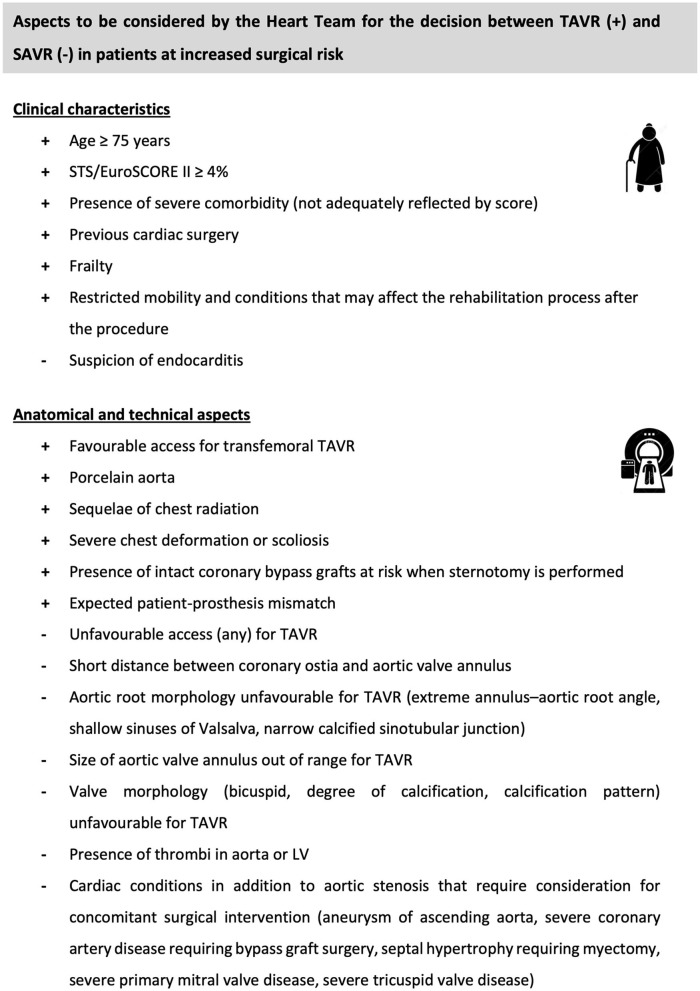
Different aspects to be considered by the Heart Team for the decision between TAVR and SAVR. SAVR, surgical aortic valve replacement; TAVR, transcatheter aortic valve replacement.

Given the current equipoise in AVR strategy that exists, patient and anatomic characteristics are critical in deciding the most favorable treatment modality. Current European Society of Cardiology (ESC) guidelines for the treatment of AS recommend SAVR for patients at low surgical risk and TAVR for patients at increased surgical risk as assessed by the Heart Team (Class 1, level C) (3). Importantly, a recommendation exists that the decision between SAVR and TAVR should be made by the Heart Team according to individual patient characteristics (Class 1, level B). Specific aspects to be considered by the Heart Team when deciding between SAVR and TAVR have been described and are reported in Figure 1. Future updates to the ESC guidelines are also anticipated such that the level of surgical risk acceptable for TAVR will be lowered.

The recently published 2020 American College of Cardiology/American Heart Association (ACC/AHA) guidelines provided insights for choice of SAVR vs. TAVR for patients with severe AS for whom a bioprosthetic AVR is appropriate (4). In patients with a high or prohibitive risk for SAVR, decision-making focuses on TAVR vs. palliative treatment. When surgical risk is not high or prohibitive, procedure-specific impediments are assessed. When both SAVR and TAVR are feasible, a prime consideration is the limited data on TAVR durability. Balance between expected patient longevity and valve durability is needed and patient age is often used as a surrogate for life expectancy. In general, SAVR is recommended for adults <65 years of age, while transfemoral TAVR is recommended for adults who are >80 years of age. For patients who are 65–80 years of age, either SAVR or transfemoral TAVR is recommended after shared decision making. Once the decision is made for SAVR or TAVR, the procedure can then be further individualized to account for specific patient characteristics or anatomical features, which may favor one approach or device over another. Aspects to be considered in this patient-tailored AVR approach are further discussed in this article.

## Patient-Tailored SAVR

A number of considerations need to be taken when deciding for a patient-tailored surgical approach. Patient factors, specific prosthesis properties and different surgical interventional techniques are the key considerations. The preference for a certain technique and/or type of prosthesis can be a combination of patient and surgeon preferences, scientific (dis)advantages as well as institutional regulations and tenders.

## Access

Some of the complications in surgery are related to the choice of access to the aortic valve. Traditional access is through median sternotomy. This access offers an excellent overview and the possibility of performing all types of surgical aortic valve intervention and even a combination of different open-heart procedures. The major risks of full sternotomy are dehiscence and sternal infection. The risk of sternal infection is 1–3% (12–14). If deep sternal infection occurs, the mortality is significant with mortality rates of 1–19% (15).

Another access to the aortic valve can be through minimal invasive surgical incisions, which has been developed since the 90's. Minimal invasive aortic valve surgery (MIAVS) access is normally either in the form of an upper hemi-sternotomy (UHS) or through a right anterior thoracotomy (RAT). Both techniques have great success if the surgical team is experienced with these minimally invasive techniques. These techniques are more surgically challenging, require more preparation and are potentially of a higher risk if not performed appropriately. On the other hand, the benefits of MIAVS are cosmetic, less pain, less bleeding, shorter duration of mechanical ventilation, shorter ICU and hospital stay as well as a faster recovery (16).

## Types of Surgical Intervention

**Figure 2:**
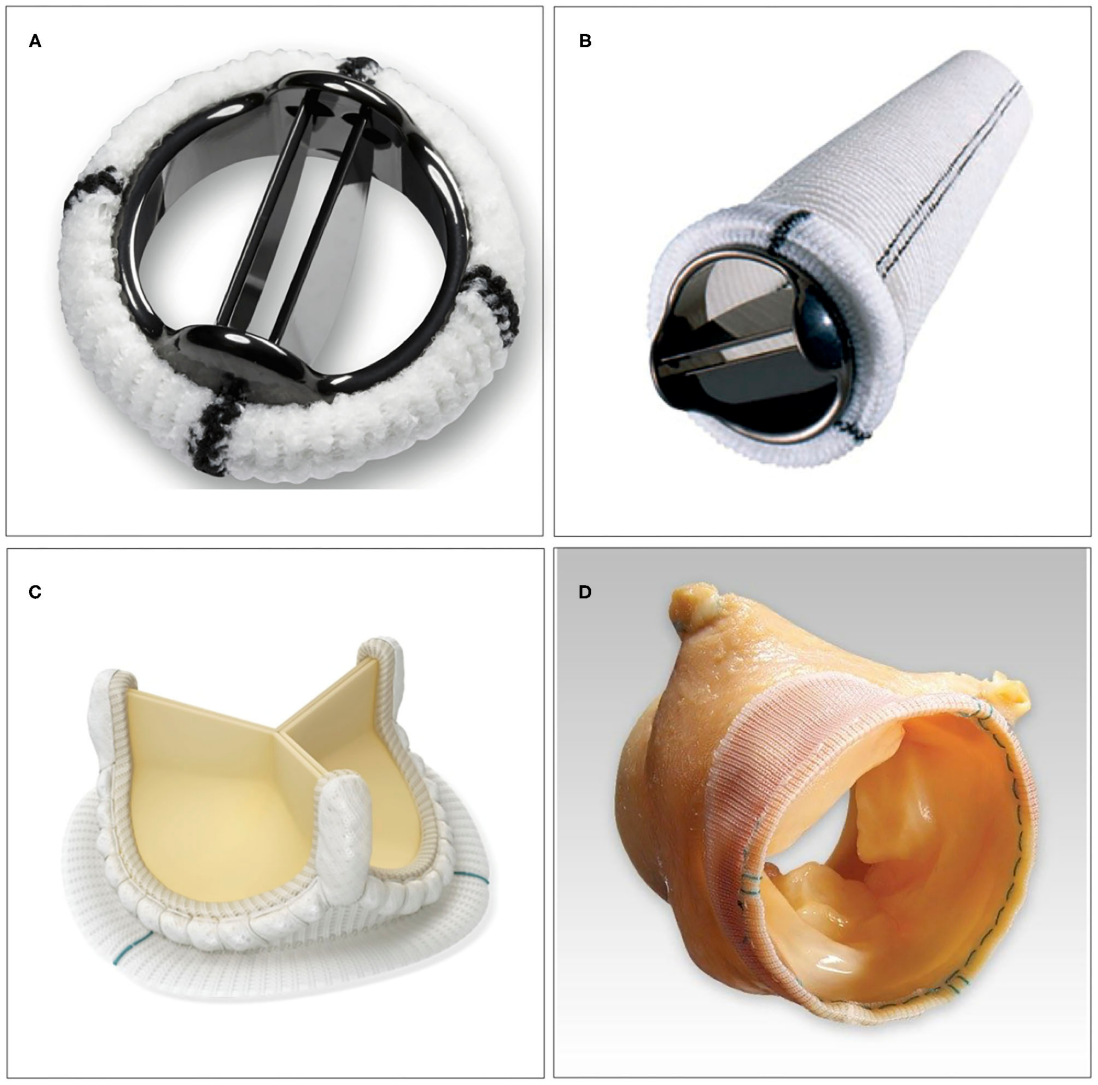
Examples of surgical aortic valve interventions. **(A)** Bi-leaflet mechanical prosthesis. **(B)** Mechanical composite graft used in aortic root replacement. **(C)** Stented bioprosthetic valve (Avalus^TM^, Medtronic, MN, USA). **(D)** Stentless bioprosthetic valve (Freestyle^TM^, Medtronic, MN, USA).

There are several factors to consider when choosing different types of aortic valve intervention. First, an assessment needs to be made whether the native valve needs to be repaired or is expected to be eligible for repair. The next choice is between mechanical vs. biological valve prosthesis, if valve replacement is needed (Figure 2). Finally, in case of a non-salvageable valve in conjunction with the need for replacement of the aortic root, a choice among different methods of root replacement needs to be made.

### Aortic Valve Repair vs. Aortic Valve Replacement

Preoperative echocardiographic assessment is essential to determine whether the patient is most suitable for aortic valve repair or replacement. AS is not repairable as shown in clinical practice in the Euro Heart Survey (17), where no cases of AS were repaired. In contrast, AR can be repaired under certain circumstances. It has been estimated that 2–8% of aortic valves can be repaired (18). Aortic valve repair is challenging and requires high-quality echocardiography as well as a dedicated and specialized surgical team. Preoperative echocardiography can identify the etiology of AR and help in planning the expected repair strategy. Repair can be performed using a multitude of different techniques. Minor repair can be done by single stitches in changing cusp prolapse, Cabrol stitching in changing commissure suspension, and patches in case cusp tissue is missing or insufficient. Major valve repair can be done by either the David (reimplantation of the aortic valve) or the Yacoub (remodeling of the aortic root) technique for replacement of the sinus of Valsalva as well as aortic annuloplasty. These techniques are well-described by Lansac et al. including objective measurements for cusp repair (19). The relatively new Ozaki technique describes a method of aortic valve reconstruction by means of replacing all three aortic cusps by glutaraldehyde-treated autologous pericardium. This technique can be performed via an upper mini-sternotomy.

AR can be grouped into three types; each of which requires different repair techniques (20, 21). All types of AR can be repaired, but type III AR and the use of pericardial patch appear to be risk factors for later reintervention (21, 22). According to current European guidelines, aortic valve repair may be a feasible alternative to valve replacement for patients with AR in a pliable, non-calcified tricuspid aortic valve or bicuspid valve regurgitation—the decision should be made following a Heart Team discussion (3). Advantages of valve repair are lower risk of thromboembolic complications compared with valve replacement, no need for anticoagulation compared with mechanical prosthesis, excellent hemodynamic performance, low mortality and less valve-related complications (18). Mid- to long-term outcomes of valve repair are comparable to replacement with biological prosthesis, but the risk of reoperation in repair appears higher than with mechanical prosthesis. In conclusion, most types of AR are treatable by valve repair, and valve repair should be prioritized over replacement in the correct clinical setting, as valve repair offers many advantages. Nevertheless, the most frequent treatment of aortic valve disease is still, by far, aortic valve replacement.

### Mechanical vs. Biological Valve Prosthesis

Either mechanical or biological types of prosthesis can be used in SAVR. Both groups consist of different subtypes. All current types of mechanical prosthesis have a bi-leaflet design. Difference in the bi-leaflet design results in difference in effective orifice area (EOA) and level of anticoagulation needed. Carbon is the material of choice for mechanical prostheses, but titanium and stainless steel are also used (23). Pannus formation is a complication primarily related to mechanical prostheses with an occurrence of 0.24%/patient per year (24). A major advantage of mechanical prostheses is their excellent long-term durability.

The main subtypes of surgical biological prosthesis are stented valves, xenografts and autografts. Stented valves can be built using either bovine pericardium or porcine aortic valve cusps. The biological material of choice is then mounted on a stented frame of metal or plastic by using woven polyester material. A sewing ring is constructed using woven polyester material and sometimes silicone rubber. The stented valves are by far the most used replacement prosthesis. Pericardial valves are evolving more than cusp-based prosthesis, so they are, in many cases, the primary choice. Novel innovations include future-proofing of surgical bioprostheses to facilitate valve-in-valve TAVR. This includes a hinged frame to enable implantation of the largest possible TAVR and fluoroscopic markers to aid TAVR sizing.

Sutureless valves are biological prostheses based on the same principle as the TAVR stent, facilitating quicker and easier implantation in a minimally invasive procedure and a shorter non-heart beating time during operation. Xenografts are often used in aortic root replacement, but not so much in simple aortic valve replacement. Indication for using xenografts in simple aortic valve replacement could be a need to achieve the largest possible valve opening area especially in patients with risk of patient-prosthesis-mismatch. The procedure requires the surgeon to re-implant the coronary arteries and is therefore more technically challenging than performing a standard SAVR. Other usages of xenografts and homografts are in re-operations or aortic valve endocarditis, as they offer pliability of the suturing line and better compensation for loss of tissue due to infection. Xenografts are easier to keep in stock and available in correct sizes. In contrast, homografts are scarce in number, need to be handcrafted individually and need special nitrogen storage. Autograft is typically used in the Ross operation, in which the pulmonary root is moved to the aortic position and the pulmonary root is replaced by a homograft (25). The pulmonary valve and aortic valve resemble each other closely and therefore the durability of the autograft is excellent. Ross operation is a complex operation that requires a dedicated surgical team and a high procedural volume to secure satisfactory results.

According to current guidelines, mechanical prosthesis is recommended for patients aged 50 years or less, for patients with longer life expectancy, for patients who are already on anticoagulants for other indications and for patients with risk of accelerated structural valve deterioration. The same guidelines recommend biological prosthesis for patients aged 65 years or more, for patients contemplating pregnancy, and for patients where anticoagulation is unlikely to be successful. The choice should also take into account the desire of an informed patient (3, 4). Center-expertise is needed to determine the use of more specialized operations such as the use of autografts or homografts.

### Aortic Root Replacement

In case there is a need for aortic root replacement, there are various surgical treatment strategies. Four possibilities have already been mentioned namely xenograft, autograft, homograft and aortic valve sparing operations (David/Yacoub/Caviaar techniques). Two other options are implantation of either a mechanical or biological composite graft consisting of both valve prosthesis and a tubular graft mounted together. The choice of valve type is normally coherent with the current guidelines as mentioned above (3, 4), but patients in need of aortic root replacement in general have more co-morbidities that one needs to take into account when choosing the valve type. A biocomposite root replacement procedure has a higher risk of reoperation than valve repair (26). When done in the correct setting, studies have shown a 10-year freedom from reoperation in 85–95% of aortic valve-sparing operations; making the long-term survival comparable to the general population (27). Compared with aortic valve replacement, aortic root replacement is a relatively larger surgical trauma. Amongst the steps in aortic root replacement, re-implantation of the coronary arteries always carries the risk of myocardial ischemia and thereby post-operative complications.

## Summary

Surgical techniques are rapidly evolving including minimally invasive techniques, increased use of valve repair techniques, better surgical prosthesis and shorter operation time. All these advancements aim at minimizing complications, ensuring quicker recovery and securing longer prosthesis durability. A dedicated Heart Team approach remains critical to delineate the most suitable and tailored strategy for patients undergoing surgical aortic valve intervention.

## Patient-Tailored TAVR

Treatment options for patients with severe symptomatic AS have expanded significantly in recent years. There is now evidence for the use of TAVR in elderly patients with severe AS across all surgical risk categories (3–11). For pure native AR, TAVR is not a guideline-recommended treatment option to date (3, 4, 28). Certain dedicated TAVR systems are currently under investigation for the usage in pure native AR.

Careful consideration of patient and anatomical characteristics is required when selecting the most suitable TAVR device. This selection process is especially important as TAVR expands into a younger patient cohort, where decisions taken for the first TAVR device will affect subsequent intervention in these patients with longer life expectancy. Co-morbidities and life expectancy of each patient should therefore be considered and discussed at the Heart Team meeting. The vital role of high-resolution computed tomography (CT) imaging of the aortic valve and peripheral vessels must also be underlined. Many complications can be minimized or avoided through meticulous pre-procedural planning.

**Figure 3:**
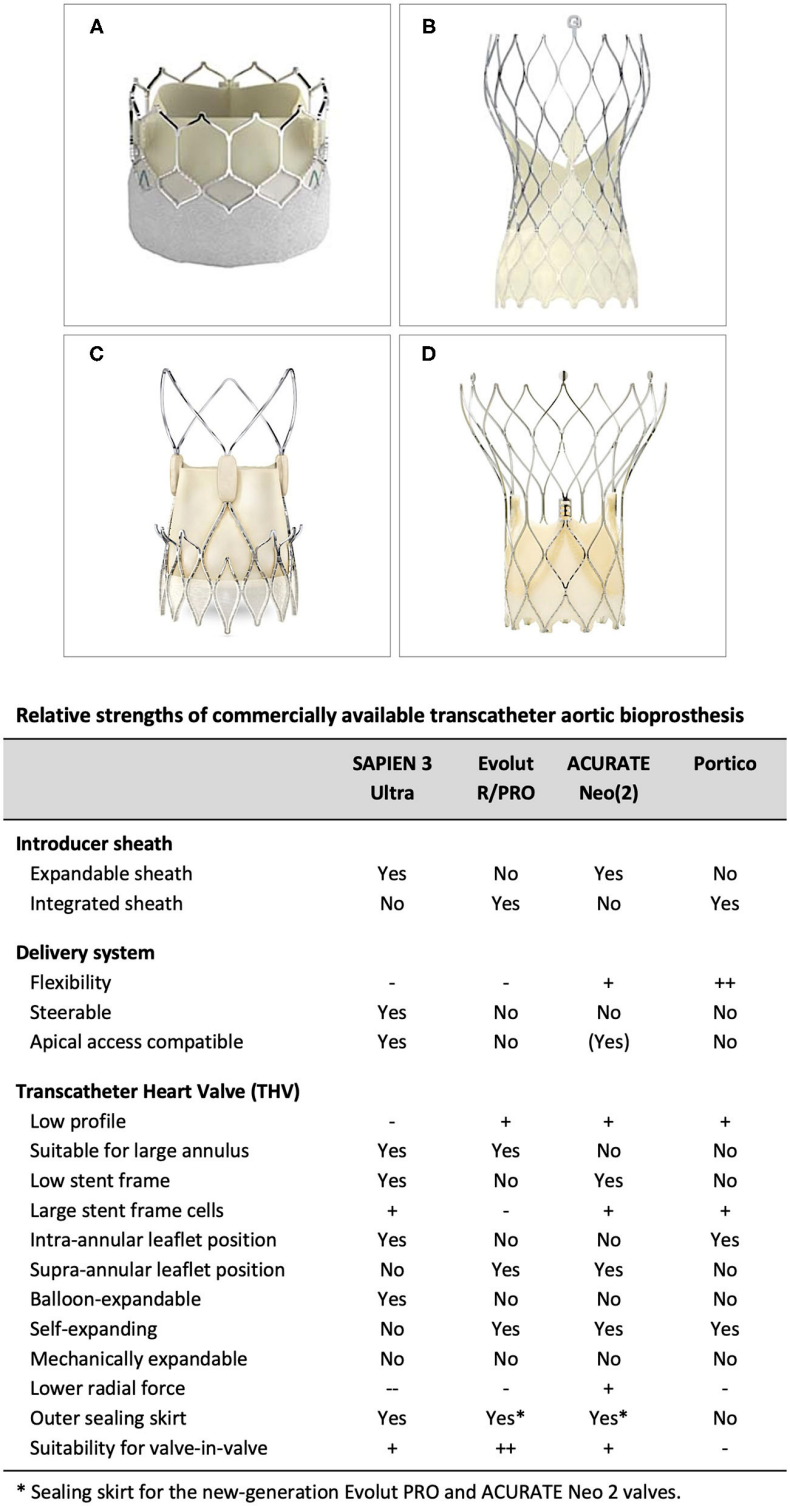
Relative strengths of commercially available transcatheter aortic bioprosthesis. **(A)** SAPIEN 3 (Ultra), **(B)** Evolut R/PRO, **(C)** ACURATE Neo (2), and **(D)** Portico TAVI System.

Currently available TAVR systems are able to treat a broad range of patients. Importantly, there are notable differences in insertion profile and vascular access, flexibility and vessel tracking, treatable annulus range, expansion force at the annulus, likelihood of permanent pacemaker implantation and intra-annular vs. supra-annular leaflet position. A detailed understanding of the individual TAVR system strengths and weaknesses is critical in order to optimize TAVR results (Figure 3). Although few head-to-head comparative studies comparing two different TAVR platforms are available - such as the SCOPE trials, comparing the ACURATE Neo valve with the balloon-expandable SAPIEN valve (SCOPE I) and self-expanding Evolut platform (SCOPE II)—these studies are not very useful, as these studies investigate the performance of these devices when used in an all-comers population. However, this is not how physicians (should) use these devices in daily clinical practice. A patient-tailored transcatheter heart valve (THV) choice is an absolute must when aiming for the best procedural and long-term outcomes for these patients. Utilizing favorable aspects of the THV and/or delivery system to minimize procedural risks and optimize outcomes is strongly recommended. Also, understanding the limitations of different TAVR systems is crucial in avoiding unnecessary procedural risks, especially in patients with challenging anatomy.

## Access

Vascular access forms a vital aspect of TAVR system selection. Significant iliofemoral disease is common in TAVR candidates, making the delivery system insertion profile and flexibility highly relevant or even driving the selection of an alternative vascular access. An iliofemoral luminal diameter ≥ 5 mm is generally acceptable for transfemoral TAVR—although tortuosity, calcifications and atheroma should also be taken into account when assessing vascular access suitability. Devices with smaller insertion profiles are available and may include an integrated sheath, thereby minimizing the required iliofemoral luminal diameter. Several manufacturers also produce expandable introducer sheaths, which are designed to radially dilate as the delivery system passes through the iliofemoral artery. Patients who were once considered not suitable for transfemoral TAVR can now also receive intravascular lithotripsy to treat calcified iliofemoral disease prior to TAVR (29). This can potentially expand the patient cohort eligible for transfemoral TAVR.

When transfemoral access is deemed unsuitable, alternative access for TAVR has to be considered. Although transapical access was the most used alternative access in the early days of TAVR, there is now a worldwide tendency to avoid transthoracic access and use percutaneous transvascular alternative access instead. Over the past few years, transaxillary, transsubclavian and transcarotid access have become the more popular alternative access routes for TAVR (30–32). Transapical TAVR may also still be needed, thereby requiring “reverse” loading of the THV within its delivery system.

## Aortic Valve and Aortic Root

Different aspects of the aortic valve and aortic root should be systematically assessed on the pre-procedural cardiac CT-scan: leaflet calcifications (severity, distribution, tricuspid vs. bicuspid), aortic annulus (size, calcification), left ventricular outflow tract (LVOT; size, calcification), sinus of Valsalva, sinotubular junction (size, calcification), coronary height, ascending aorta angulation.

Measurement of the aortic annulus and aortic root are the most important determinants of THV sizing. There is significant room for variation in device selection within this guide. Large annulus measurements (area > 573 mm^2^, perimeter > 85 mm) are within the treatable range of only two currently available TAVR systems (Sapien 29 mm, Edwards Lifesciences, CA, USA, and Evolut R 34 mm, Medtronic, MN, USA), although large THV sizes are also under development for other TAVR systems. TAVR has been performed in a small number of patients with aortic annulus measurements exceeding recommended limits (area up to 852 mm^2^) with good results (33). For this off-label use, long term outcomes are not known, and prosthesis durability may be affected due to excessive deformation of the THV.

There is also a consensus among TAVR experts that THVs with supra-annular leaflet position should be considered when treating very small aortic annulus size or surgical aortic bioprosthesis with a small inner diameter during valve-in-valve TAVR, thereby maximizing the aortic valve EOA. This can also reduce transvalvular gradient, which is important in improving prosthesis longevity.

In general, balloon expandable THVs are best avoided in cases with severely calcified LVOT and/or sinotubular junction with small dimensions, given the potential risk of annulus rupture and aortic dissection. Similarly, isolated large calcifications at the aortic annulus may cause excessive trauma with balloon expandable THVs.

A heavily or asymmetrically calcified annulus and LVOT should be treated with THVs having an outer sealing skirt to reduce the risk of paravalvular leak (PVL). Consideration should also be given to the higher risk of some THV systems to induce atrioventricular conduction disturbance. However, it is important to realize that the choice of THV type/size and implantation depth is a complex interplay that will determine the final risk of PVL and conduction disturbance (34).

Low coronary ostia (defined as originating ≤ 10 mm above the aortic annulus) or the need for future percutaneous coronary interventions favor the use of THVs with a low stent frame and/or intra-annular leaflets, thereby minimizing potential interference with future coronary access (35).

A horizontal aorta (aortic angulation > 60°) favors a flexible or steerable delivery system, ensuring alignment of the TAVR system with the native annulus and simplifying THV deployment. Similarly, a high degree of maneuverability can be used to navigate an acutely angulated aortic arch or descending aorta.

In patients with symptomatic severe bicuspid AS, TAVR may be considered an alternative to SAVR, provided that there is no concomitant significant aortopathy (which is not uncommon in patients with a bicuspid aortic valve). Patients with bicuspid anatomy have been excluded from the early randomized trials that have introduced TAVR into mainstream practice, although early results from large registry data show good TAVR performance in bicuspid patients with newer generation devices (36). Careful THV selection and sizing to account for asymmetric valve constriction and the greater risk for PVL, annulus rupture, and pacemaker will likely optimize TAVR results in this specific patient population.

Pure native AR has been treated with TAVR in an off-label use setting. Risks of TAVR in pure native AR include THV migration and significant PVL (28). For most patients, referral to cardiac surgery is the most appropriate decision. The JenaValve (JenaValve Technologies Inc., CA, USA) has been designed to anchor on the native valve leaflets rather than the annulus and received CE mark approval for the treatment of native pure AR. This device is currently under investigation in a clinical study for transfemoral delivery. The J-Valve (JC Medical Inc., A, USA) is another device that may treat native AR as well as AS and is currently being evaluated in an early feasibility trial.

## Patient Characteristics

Patient characteristics are important aspects that should be considered for TAVR customization as well. For instance, for patients with pre-existing conduction disturbance (e.g., right bundle branch block), selection of a THV with a lower risk of permanent pacemaker and a relatively high THV implantation should be considered (37–39). The expansion of TAVR into patients with longer life expectancy also means that the overall patient lifetime management should be taken into consideration. Early device safety will remain a key determinant of immediate success. Additionally, THV durability, long-term effects of conduction disturbance, and future coronary access are important aspects of lifelong care. For relatively younger patients, planning on a strategy for potential TAVR-in-TAVR should also be considered, as life expectancy likely exceeds THV durability in these patients. An initial THV with intra-annular leaflet position is less likely to cause coronary obstruction or issues with coronary access in case of THV-in-THV (40). Comparative studies investigating the impact of THV choice on these issues require longer-term follow up than current literature provides.

## Conclusions

Contemporary surgical and transcatheter aortic valve interventions offer effective therapy for a broad range of patients, anatomies and operative risk categories. Both approaches have seen significant advances in recent years. SAVR remains an important treatment modality for younger patients and patients with pure AR, necessity of aortic root replacement or endocarditis. Guidelines have previously emphasized ‘surgical risk’ in the decision between SAVR and TAVR, although this delineation becomes increasingly obsolete with more evidence on the effectiveness of TAVR in low surgical risk candidates. More importantly, decisions in tailoring aortic valve replacement should be patient-centered, accounting not only for operative risk, but also anatomy, lifetime management and specific co-morbidities. For both surgical and transcatheter interventions, a wide variety of procedural techniques are described. Operator experience and detailed knowledge of the limitations of these different available technologies can certainly optimize patient outcomes.

## Author Contributions

All authors contributed to the design, writing, and revision of the manuscript.

## Conflict of Interest

The authors declare that the research was conducted in the absence of any commercial or financial relationships that could be construed as a potential conflict of interest.
